# The founding of the Centre for Palliative Medicine, Medical Ethics and Communication Skills: a new step toward the development of patient-oriented medicine in Croatia

**DOI:** 10.3325/cmj.2011.52.87

**Published:** 2011-02

**Authors:** Veljko Đorđević, Marijana Braš, Vibor Milunović, Lovorka Brajković, Ranko Stevanović, Ozren Polašek

## Patient-oriented medicine

One of the main conceptual changes in the 20th century medicine is the inclusion of social dimension. The “golden era” of Parson’s medical model (1), which uses the “active-passive” dichotomy to describe the positions and expectations of physicians and patients, is over. Physicians’ supremacy has slowly and systematically been challenged by the emergence of third party stakeholders, development of new media sources, strengthening of the civil society, and democratization of information, which all have contributed to the development of the patients’ active role in the healing processes (2). The rise of medical consumerism has stimulated the medical authorities to react with a new ideological policy: the patient-oriented medicine, insisting on the partnership in the diagnostic and therapeutic processes, and viewing the patient as a person with biological, psychological, social, and spiritual needs (2). This goal has been developing in the Western societies for years, while it is still insufficiently recognized in Croatia. There are several indicators of this problem in Croatia: the insufficient and unsystematic institutional approach to palliative medicine, weak non-governmental organization network, misrecognition of the importance of communication processes in everyday physicians’ practice, insufficient education beyond the mechanistic approach to disease, and inadequate legislative practice dealing with patients’ rights.

## The form and structure of the Centre

As a reaction to the negative situation in the Croatian medical system, the Centre for Palliative Medicine, Medical Ethics and Communication Skills (CEPAMET) was founded on September 21, 2010. The Centre is semi-autonomous unit within the School of Medicine, University of Zagreb. The conceptual framework of the Centre is devoted to education, organization, and research in three domains: palliative medicine, ethics, and health communication. The interdisciplinary team involved in the CEPAMET creation consists of experts in psychiatry and psychology, neurology, oncology, and general practice. The Centre also has an advisory board, consisting of the most prominent international and national experts.

## Palliative medicine

Despite the obvious lack of the palliative medicine facilities and overall positive attitudes in the general public (3,4), palliative medicine has until now mostly belonged to the domain of volunteer work and non-governmental organizations. The CEPAMET is the first institutionalized attempt to systematically include palliative medicine into the system of medical care. As Professor Anica Jusić, a pioneer in the hospice movement in Croatia, stated in her overview of palliative medicine, we are entering the 4th era after the failure to implement palliative care in the institutional medicine (5). As a response to this failure, the CEPAMET's main short-term goal is to develop reasonable, scientifically based, and cost-effective national strategy for palliative medicine, leading to specific initiatives for its implementation at the primary, secondary, and tertiary health care level. The long-term goal is to reorganize some of the existing health care capacities into the stationary palliative care hospital wards. The CEPAMET’s professional plan is to provide an educational basis for palliative care, ie, introduce obligatory subjects in undergraduate and postgraduate curriculum, with a purpose to create a new specialty in the Croatian health care system, that of palliative care. The education plan includes various courses devoted to communication with oncologic patients and a postgraduate course on pain and palliative medicine to be held in the International University Centre in Dubrovnik. The research plan includes conducting research studies that will aim to identify the status and obstacles to further development of this field, explore cultural determinants of palliative medicine, patients’ and physicians’ needs, and measure the outcome of specific interventions in order to plan and perform interventions.

## Communication skills

Proper communication skills have often been neglected in medical education in Croatia, but in the EU and USA there is an emphasis on communication in different, often tricky, situations that physicians encounter on a daily basis. The CEPAMET will offer education in these skills to both undergraduate and postgraduate students, as well as other professionals. Several CEPAMET units are constructed as communication skills laboratories, where students and professionals will have a chance to practice with real-life and simulated patients using video cameras and scales, such as Roter Interaction Analysis System and Cambridge Guide to Medical Interview (6,7). This will give proper up-to-date tools to evaluate, modify, and adopt various aspects of communication processes.

Several members of CEPAMET have already been assigned a role in the Teaching Committee of the European Association for Communication in Health Care. Despite numerous studies in the area of medical communication, the evidence-based outcomes of communication interventions are still largely unclear (8). The development of communication skills teaching within CEPAMET will include planning a wide range of studies, involving social, emotional, and health outcomes in order to contribute to this scientific field.

## Medical ethics

The third area of interest of the CEPAMET is medical ethics. With the progressive social and technological changes in the Croatian medicine, the issue of ethics, ethical conduct, and ethical problems is more important than ever. To deal with this problem, several guidelines are being planned to improve ethical conduct and avoid possible ethical pitfalls in the everyday medical practice. Medical ethics principles are often neglected in education and cognitive processes. The CEPAMET strives to popularize ethics in medical community and educate professionals in current opinions and philosophical theories regarding the issue. Furthermore, education is meant to be focus-oriented, guiding physicians and students through the complex and commonly problematic world of applied ethics, as opposed to teaching and proposing theoretical stance.

## Is CEPAMET enough?

Although CEPAMET is certainly an ambitious project, it is only a small step toward improving our medical reality. Without substantial support from various levels of our health care system, academic community, civil society, and patients, CEPAMET’s goals are unlikely to be met. Therefore, we would also like to offer a possibility of collaboration to both national and international partners, in order to further contribute to the development of patient-centered medicine in Croatia.

## References

[1] Parsons T. The social system. New York (NY): The Free Press; 1964.

[2] Mead N, Bower P (2000). Patient-centredness: a conceptual framework and review of the empirical literature.. Soc Sci Med.

[3] Brkljacic M, Mavrinac M, Sorta-Bilajac I, Bunjevac I, Cengic T, Golubovic V (2009). An increasing older population dictates the need to organise palliative care and establish hospices.. Coll Antropol.

[4] Andrijevic-Matovac V (2010). The light from Bethlehem.. Croat Med J.

[5] Jusić A (2009). The hospice movement in Croatia – short review. Acta Med Croatica.

[6] Roter D, Larson S (2002). The Roter Interaction Analysis System (RIAS): utility and flexibility for analysis of medical interactions.. Patient Educ Couns.

[7] Silverman JD, Kurtz SM, Draper J. Skills for Communicating with Patients. Oxford (UK): Radcliffe Medical Press; 1998.

[8] de Haes H, Bensing J (2009). Endpoints in medical communication research, proposing a framework of functions and outcomes.. Patient Educ Couns.

